# Visual Impairment among Older Adults in a Rural Community in Eastern China

**DOI:** 10.1155/2016/9620542

**Published:** 2016-09-29

**Authors:** Chen-Wei Pan, Deng-Juan Qian, Hong-Peng Sun, Qinghua Ma, Yong Xu, E. Song

**Affiliations:** ^1^Jiangsu Key Laboratory of Preventive and Translational Medicine for Geriatric Diseases, School of Public Health, Medical College of Soochow University, Suzhou 215123, China; ^2^The 3rd People's Hospital of Xiangcheng District, Suzhou 215134, China; ^3^Lixiang Eye Hospital of Soochow University, Suzhou 215021, China; ^4^Department of Ophthalmology, The First Hospital of Jilin University, Changchun 130021, China

## Abstract

*Purpose*. To determine the prevalence, causes, and associations of visual impairment (VI) among participants aged 60 years or older in a rural community in China.* Methods*. A community-based survey was undertaken in a rural town located in Eastern China and 4579 people aged 60 years or older participated in the study. Presenting visual acuity was assessed using a Snellen chart with tumbling-E optotypes and anterior segment was examined using a slit-lamp. VI was defined as presenting VA <6/18 and it included moderate VI (<6/18 to 6/60) and blindness (<6/60).* Results*. The prevalence of VI was 5.4% (95% confidence interval [CI] 4.7–6.0). In multivariate analysis, the presence of VI was positively associated with increasing age (odds ratio [OR] = 1.12, 95% CI 1.10–1.16, per year increase), female gender (OR = 2.33, 95% CI 1.53–3.55), the presence of hypertension (OR = 1.31, 95% CI 1.001–1.85), living alone (OR = 1.52, 95% CI 1.08–2.62), and increased sleeping hours (OR = 1.10, 95% CI 1.001–1.22). Drinking 3 or more glasses of green tea per day was inversely associated with VI (OR = 0.79, 95% CI 0.63–0.98).* Conclusion*. VI was less prevalent in this community compared with previous report in other areas in China.

## 1. Introduction

Visual impairment (VI) is a devastating disability worldwide [1–7] and has been reported to be linked with functional limitations [8], falls [9], depression [10], cognitive function [11], and mortality [12, 13]. The health burden associated with VI has been estimated to be heavier than hypertension, diabetes, obesity, and hyperlipidemia [14]. The “Vision 2020: The Right to Sight” proposed by the World Health Organization has reduced 15 million cases of blindness globally [15]. Recently, the World Health Organization's report on “universal eye health: a global action plan 2014–2019” indicated that many people with VI were still undetected or untreated. Therefore, it is important to conduct periodic regional epidemiologic studies to understand both the burdens of VI and plan strategies to address this issue.

China is the world's most populous country. The prevalence of VI in China is estimated to be 10% in adults aged 50 years or older [1]. Understanding the epidemiology of VI in China may have considerable public health implications. Although quite a few studies [16–21] have reported the prevalence and possible causes for VI in the mainland of China, these findings cannot be directly extrapolated to other areas considering the huge disparities in environmental exposures, cultures, socioeconomic status, lifestyle-related factors, and healthcare systems in China.

We undertook a community-based study in a rural town in Suzhou, China. People living here have a long history of drinking green tea habitually. It has been suggested that phytochemicals such as green tea catechin may serve as antioxidants and anti-inflammatory agents and help prevent or delay the progression of age-related eye diseases such as cataract, glaucoma, and age-related macular degeneration [22]. Whether this specific lifestyle-related exposure could contribute to a low prevalence of VI remains unclear. In this analysis, we report the prevalence, possible causes, and associated factors of VI among older adults aged 60 years or older in this community.

## 2. Materials and Methods

### 2.1. Study Cohort

The Weitang Geriatric Diseases study was a community-based survey conducted in the Weitang town located in Suzhou in Eastern China. The Weitang town has a relatively stable population and is in a lower socioeconomic status compared with many other towns in Eastern China. Residents in this town are poorly educated and quite a few of them were farmers. There is only one hospital which provides eye healthcare service for the residents in this town. This study was conducted from 2013 to 2014. The aim of the study was to estimate the patterns, predictors, and burden of common health outcomes including chronic disorders (e.g. hypertension, diabetes, and hyperlipidemia), cognitive dysfunction, depressive symptoms, and health-related quality of life of elderly people aged 60 years or older in this area. Every subject underwent standardized systematic and ocular examinations, interviewer-administered questionnaires, and blood investigations for risk factor assessment. Based on official records, 6,030 names aged 60 years or older resided in the town. Before the study, an invitation letter was sent to each family and nature of the study was explained in the letter. All the adults aged 60 years or older in the town were invited to participate in this study. An adult was considered “ineligible” to participate if he or she had moved from the residing address, had not been living there for more than 6 months, or was deceased. Of the 6,030 names listed in the official records, 5,613 subjects were considered to be “eligible” to participate in this study. In the end of the study, 4,611 elderly adults (82.1%) participated in eye examinations. Compared with nonresponders, responders of this study were younger (67.6 versus 71.4 years; *P* = 0.01), but there were no gender differences (*P* = 0.52).

The Weitang Geriatric Diseases study was conducted following the tenets of the Helsinki Declaration and was approved by the Institutional Review Board of Soochow University. The study methods were carried out in accordance with the approved guidelines. All participants gave written informed consent at the recruitment stage of the study.

### 2.2. Eye Examinations

Eye examinations were part of the study and each participants underwent visual acuity (VA) measurement, anterior segment examination, autorefraction, and distant direct ophthalmoscopy. VA was measured in both eyes by trained research optometrists using a retroilluminated Snellen chart with tumbling-E optotypes (Precision Vision, La Salle, IL, USA) at a distance of 4 m. The presenting VA (PVA) was recorded with the participant wearing his or her habitual optical correction such as spectacles or contact lenses, if any. If no number could be identified at 4 m, the participant was moved to 3, 2, or 1 m, consecutively. If no number could be identified at all, VA was examined as counting fingers, hand movements, perception of light, or no perception of light. Moderate VI was defined as presenting VA worse than 6/18 to 6/60 in the better-seeing eye and blindness was defined as presenting VA worse than 6/60 in the better-seeing eye. In this study, VI was used as generic term including both moderate VI and blindness.

The case definitions for the causes of VI were similar to the Rapid Assessment of Visual Impairment (AP-RAVI) project in India [23]. Cataract was defined according to the Lens Opacities Classification System (LOCS) III [24]. Cataract was considered as the main cause of VI if there was no evidence of retinal abnormality in an eye with significant cataract that obscured the vision. Uncorrected refractive error was defined as presence of presenting VA worse than 6/18 and improving to 6/18 or better with a pinhole. Posterior segment diseases were considered as the cause of VI in cases where there was no media opacity and VA did not improve with a pinhole. The causes of VI were first recorded for each eye separately and then mapped to the person. Where there was more than one cause, the condition that could be most easily corrected or treatable was considered as the cause for VI [25].

### 2.3. Measurement and Definitions of Risk Factors

At interview, data were collected on education level (no formal education/primary education/high school/university) and lifestyle-related factors such as number of hours for outdoor activities and sleeping per day. Other data included smoking status (current/past/never smoked), alcohol intake within the past 3 months (yes/no), and green tea consumption for least one year (yes/no) and whether participants had previously been diagnosed with diabetes or hypertension by a doctor. In addition, the amount of smoking, alcohol intake, and green tea consumption per day were recorded if the answer related to these questions was “yes.” Systolic, diastolic blood pressure and pulse rate were measured using an automated blood pressure monitor, following the protocol used in the Multiethnic Study of Atherosclerosis [26]. Fasting venous blood samples were collected and sent for biochemistry tests, including analysis of cholesterol, triglycerides, glucose, and creatinine. Diabetes mellitus was defined as fasting glucose levels of more than 7.0 mmol/L or physician diagnosis of diabetes or use of diabetic medications [27]. Hypertension was defined as systolic blood pressure of 140 mmHg or more or diastolic blood pressure of 90 mmHg or more, physician diagnosis of hypertension, or use of antihypertensive medications.

### 2.4. Statistical Analyses

Statistical analyses were performed using STATA version 11.0 (StataCorp, College Station, TX, USA). Prevalence estimates and 95% confidence intervals (CIs) of VI or blindness were calculated, either stratified by age and gender or as a whole. The age-standardized prevalence was calculated by direct standardization of the study samples to the population in Suzhou, using the 2010 national census data (http://www.stats.gov.cn/). The prevalence estimate was compared with other population-based studies conducted in rural China. A multinomial logistic regression model was used to assess the risk factors associated with the presence of VI. Only age, gender, and variables with *P* value of less than 0.10 in univariate models were included in multiple logistic regression models and odds ratios (ORs) and 95% CI were calculated.

## 3. Results

Of the 4611 participants of this study, 4579 had reliable VA data and were included in the analysis. 2200 (48%) were male and 2379 (52%) were female. The age ranged from 60 to 93 years with mean of 67.6 ± 6.3 years. The mean ages of men and women were 67.5 ± 6.1 and 67.8 ± 6.5, respectively (*P* = 0.08). Of the 4579 people included in the analysis, 82 (135 eyes) had undergone cataract surgery.

Among all the study participants, 525 had VI and 44 had blindness.  Table 1 shows crude and age-standardized prevalence of VI and blindness by age and gender. The age-standardized prevalence of VI was 5.4% (95% CI 4.7–6.0) and was significantly higher in women (7.4%; 95% CI 6.3–8.4) compared with men (3.2%; 95% CI 2.5–3.9) (*P* < 0.001). VI affected 1.6%, 2.8%, 5.1%, 10.1%, and 19.7% of men aged 60 to 64 years, 65 to 69 years, 70 to 74 years, 75 to 79 years, and 80 years or older and 2.6%, 6.9%, 10.7%, 20.2%, and 33.3% of women in same age groups, respectively. The increasing prevalence of VI with increasing age was statistically significant in both men and women (*P* for trend < 0.05). With regard to blindness, the overall age-standardized prevalence was 0.45% (95% CI 0.26–0.64). The prevalence of blindness was also higher in women than in men (0.73% versus 0.14%; *P* < 0.001).

Table 2 shows the distribution of causes of moderate VI and blindness in this community. Cataract was the leading cause of blindness which accounted for 72.7% of the total cases with blindness, while uncorrected refractive error was the leading cause of moderate VI (46.6%). Other causes of moderate VI included posterior segment disorders (3.5%), corneal opacity or pterygia (6.4%), and cataract surgical complications (0.8%). Other causes of blindness included posterior segment disorders (18.2%) and absent globe (9.1%).

Table 3 demonstrates the associations between potential risk factors and the presence of VI. In univariate analysis, age, gender, education, monthly income, hypertension, living arrangements, smoking, alcohol intake, green tea consumption, sleeping hours per day, and time spent outdoors per day were significant factors (all *P* < 0.10). In multivariate analysis, the presence of VI was positively associated with increasing age (OR = 1.12, 95% CI 1.10–1.16, per years increase), female gender (OR = 2.33, 95% CI 1.53–3.55), the presence of hypertension (OR = 1.31, 95% CI 1.001–1.85), living alone (OR = 1.52, 95% CI 1.08–2.62), and increased sleeping hours (OR = 1.10, 95% CI 1.001–1.22). Drinking 3 or more glasses of green tea per day was inversely associated with VI (OR = 0.79, 95% CI 0.63–0.98). In addition, we performed separate analysis on the association between green tea consumption and VI associated with cataract and refractive error, respectively. The association between green tea consumption and VI associated with cataract remained significant (OR = 0.84, 95% CI 0.69–0.99). The association between green tea consumption and VI associated with refractive error was not significant (*P* = 0.57).

## 4. Discussion

In this community-based study of older adults, we reported a relatively lower prevalence estimate of VI compared with previous reports in mainland of China. Based on PVA, only 6.7% of the study participants were affected by VI and 0.52% were blind. As expected, cataract and uncorrected refractive error were the two principal causes of VI. Increasing age, female gender, the presence of hypertension, living alone, sleeping hours, and green tea consumption were significant associated factors.

Comparing prevalence estimates among different populations should account for methodological disparities among different studies such as sampling strategies, definition of the outcomes, and age range of the study subjects. Based on presenting VA data, the prevalence of VI was lower compared with previous reports in mainland of China. For example, prevalence of (VI including blindness) was 16% and 19% in Chinese adults aged 50 years or older in Hainan [28] and Yunnan [19] province, respectively. Cataract surgery is an important contributing factor for the prevalence of VI. The prevalence of cataract surgery in our study population was not higher than that in the Hainan and Yunnan studies. Thus, cataract surgery rates were unlikely to explain the low prevalence of VI in this population. The Vision Loss Expert Group of the Global Burden of Disease Study reported that VI affects about 10% of the people aged 50 years or older in China. Considering that our study participants were even older (60 years or older), the burden of VI was much lower than Chinese living in other areas. The prevalence was also lower compared with other countries [2–5, 7]. The explanation for this low prevalence estimate was unclear since our study was observational in nature and lacked a comparison group to make a cogent conclusion. Many observed and unobserved factors may contribute to this low prevalence. Based on the survey data, about 60% of the study participants had habitually drunk green tea and the mean age of beginning to drink green tea was 28 years. Green tea contains huge amount of polyphenols, which display strong antioxidant activity and anti-inflammatory effects. Both oxidative stress [29, 30] and inflammation [31] have been strongly implicated in the pathophysiology of vision-threatening eye diseases such as age-related macular degeneration, diabetic retinopathy, and cataract. Our previous case-control study has indicated that green tea is protective for diabetic retinopathy [32]. However, this is just a hypothesis and should be verified by future studies. In addition to green tea consumption, low prevalence of VI may also be attributed to other population-wide changes in environmental variables which may not be captured in this study.

Besides green tea consumption, visually impaired adults tended to have hypertension, live alone, and sleep for more hours per day. Hypertension is one of the most common systemic chronic disorders among older people and has profound impacts on the eye. Hypertension has been directly and indirectly linked with a wide spectrum of vision-threatening eye disorders including hypertensive retinopathy, retinal vein occlusion, ischemic optic neuropathy, diabetic retinopathy, age-related macular degeneration, and glaucoma, which could all have impaired the vision [33]. In view of the significant association between hypertension and VI, we recommend that fundoscopy examinations should be integrated in the daily clinical management of hypertensive patients and physicians are encouraged to make a referral to an ophthalmological consultation when necessary.

Individuals who live alone were more vulnerable to VI in our study. This finding was consistent with previous studies which found that unmarried status is an independent risk factor for VI [34]. A possible explanation for this observation is that individuals who live alone may have greater difficulties and economic pressures that kept them away from eye care services. They also lack care from the family members. Our findings indicated that living arrangements should be taken into consideration when providing social support and developing prevention strategies of VI for older people.

Sleeping is an important lifestyle-related exposure. Previous studies have shown that sleeping is associated with eye disorders such as refractive errors [35] and optic neuropathy [36]. It was interesting to find that adults with VI tended to sleep for more hours compared with those without. Sleeping for one more hour per day was associated with a 10% increased risk of VI in our study. Current evidence supporting the role of sleeping hours in the development of eye diseases or impaired vision is insufficient. This causal relationship between sleeping durations and VI may not be straightforward and may be a mixture of causal effect in both directions. Visually impaired adults may also tend to sleep for more hours as they were restricted to perform activities in daily lives.

Although this study had several strengths, including a large and representative sample and the using of a standardized protocol to measure visual acuity, there were still some limitations, which need to be acknowledged. First, we only measured presenting VA and best-corrected VA was not measured in this study. Thus, the burden of VI excluding the effect of uncorrected or undercorrected refractive error was unclear. The reason for not collecting best-corrected VA data was that this study was not originally designed as an epidemiologic study specifically focusing on eye diseases and there were merely logistical limitations (e.g., not enough time during testing). In addition, the study protocol and the definitions used to assign causes of VI tended to overestimate the prevalence of cataract and refractive errors and underestimate the prevalence of posterior segment diseases such as glaucoma and retinal diseases. Although the causes of VI may be prone for misclassification of causes due to the use of these definitions, the prevalence of VI in itself may not be influenced by this methodology. Furthermore, participants were younger than nonparticipants, which might have underestimated the prevalence due to the increasing trend of VI with increasing age. Finally, it was a cross-sectional design so that we cannot separate cause from effect when examining risk factors.

In conclusion, the prevalence of presenting VI among older adults in this rural community in China was low. Further studies are warrant to identify the causes which could explain the low prevalence.

## Figures and Tables

**Table 1:** Prevalence of visual impairment and blindness by age and gender.

	*N*	Visual impairment (including blindness)			Blindness		
		*n*	%	95% confidence interval	*n*	%	95% confidence interval
*All (crude)*	4579	310	6.77	6.04–7.50	24	0.52	0.31–0.73
*All (age-standardized)*			5.38	4.72–6.03		0.45	0.26–0.64
*Men (crude)*	2200	95	4.32	3.47–5.17	4	0.18	0–0.36
*Men (age-standardized)*			3.19	2.45–3.92		0.14	0–0.29
*Women (crude)*	2379	215	9.04	7.88–10.19	20	0.84	0.47–1.21
*Women (age-standardized)*			7.35	6.30–8.40		0.73	0.39–1.07
*Men*							
60–64 years	898	14	1.56	0.75–2.37	0	0	—
65–69 years	606	17	2.81	1.49–4.12	1	0.17	0–0.49
70–74 years	351	18	5.13	2.81–7.45	3	0.85	0–1.82
75–80 years	228	23	10.09	6.15–14.03	0	0	—
80 years or older	117	23	19.66	12.35–26.97	0	0	—
*Women*							
60–64 years	915	24	2.62	1.59–3.66	3	0.33	0–0.7
65–69 years	714	49	6.86	5.00–8.72	6	0.84	0.17–1.51
70–74 years	327	35	10.7	7.33–14.07	5	1.53	0.19–2.87
75–80 years	258	52	20.16	15.23–25.08	2	0.78	0–1.85
80 years or older	165	55	33.33	26.06–40.6	4	2.42	0.05–4.8

**Table 2:** Distribution of causes for visual impairment and blindness.

	Visual impairment (excluding blindness)						Blindness					
	All (*n* = 481)		60–69 years (*n* = 98)		70 years or older (*n* = 383)		All (*n* = 44)		60–69 years (*n* = 7)		70 years or older (*n* = 37)	
	*n*	%	*n*	%	*n*	%	*n*	%	*n*	%	*n*	%
Cataract	205	42.6	36	36.7	169	44.1	32	72.7	4	57.1	28	75.7
Uncorrected refractive error	224	46.6	41	41.8	183	47.8	0	0	0	0	0	0
Posterior segment disorders	17	3.5	9	9.2	8	2.1	8	18.2	2	28.6	6	16.2
Corneal opacity or pterygia	31	6.4	16	16.3	15	3.9	0	0	0	0	0	0
Cataract surgical complications	4	0.8	0	0	4	1	0	0	0	0	0	0
Absent globe	0	0	0	0	0	0	4	9.1	1	14.3	3	8.1

**Table 3:** Univariate and multivariate of the associated factors for visual impairment.

	Univariate			Multivariate		
	OR	95% CI	*P*	OR	95% CI	*P*
Age (per year increase)	1.15	1.13–1.17	<0.001	1.12	1.10–1.16	<0.001
Gender (female versus male)	2.20	1.72–2.82	<0.001	2.33	1.53–3.55	<0.001
Education (no formal versus formal education)	2.19	1.72–2.79	<0.001	1.02	0.75–1.34	0.95
Monthly income (less than 1000 versus 1000 or more)	2.43	1.86–3.16	<0.001	0.95	0.69–1.48	0.32
Hypertension (yes versus no)	1.46	1.09–1.95	0.01	1.31	1.001–1.85	0.04
Diabetes (yes versus no)	1.06	0.69–1.64	0.78		—	
Living arrangements (living alone versus living with others)	1.82	1.31–2.53	<0.001	1.52	1.08–2.62	0.02
Smoking for at least 5 years (yes versus no)	1.98	1.56–3.37	<0.001	1.13	0.85–1.77	0.46
Alcohol intake within the past 3 months (yes versus no)	0.56	0.41–0.78	<0.001	0.80	0.56–1.92	0.35
Green tea consumption per day (3 glasses or more versus less than 3 glasses)	0.42	0.27–0.85	0.005	0.79	0.63–0.98	0.02
Sleeping hours per day (per hour increase)	1.48	1.37–1.59	<0.001	1.10	1.001–1.22	0.04
Time spent outdoors per day (per hour increase)	0.92	0.86–0.99	<0.001	0.93	0.86–1.22	0.27

OR: odds ratio; CI: confidence interval.
